# The impact of visual search in Chinese children with developmental dyslexia on reading comprehension: the mediating role of word detection skill and reading fluency

**DOI:** 10.3389/fpsyg.2024.1437187

**Published:** 2024-11-20

**Authors:** Hu Meng, Ru Yao, Pan Zhang, Jialei Wang, Yajing Zhang

**Affiliations:** ^1^Department of Psychology, Hebei Normal University, Shijiazhuang, China; ^2^China National Academy of Educational Sciences, Beijing, China

**Keywords:** developmental dyslexia, visual search, word detection skill, reading fluency, reading comprehension

## Abstract

**Introduction:**

Previous research has highlighted the influence of visual search (VS) on reading comprehension (RC); however, the underlying mechanisms of this effect remain poorly understood, particularly in children with reading disabilities. This study explores disparities in VS, word detection skills (WD), reading fluency (RF), and RC between Chinese children with developmental dyslexia (DD) and their typically developing peers across different age groups.

**Methods:**

The sample comprised 191 students from grades 2, 4, and 6, including 92 children with dyslexia and 99 chronological age-matched controls. Variance analysis was used to examine differences in VS, WD, RF, and RC performance. Structural equation modeling (SEM) was employed to assess the relationships among these variables.

**Results:**

Children with DD performed significantly worse than their typically developing peers on VS and RC tasks, with the most notable differences emerging in the middle and higher grades. Additionally, children with DD showed weaker WD skills and RF, with these disparities evident across all grade levels. SEM indicated that VS directly influences RC, with WD and RF serving as mediators in the relationship between VS and RC.

**Discussion:**

These findings elucidate the complex interplay between visual processing and linguistic skills in reading development, particularly within the Chinese language context. The study underscores the importance of targeted interventions for children with dyslexia, emphasizing strategies that address the unique challenges these learners face in Chinese reading environments.

## 1 Introduction

Developmental Dyslexia (DD) is a prevalent learning disorder observed in children who, despite possessing normal intelligence and lacking visible neurological or organic impairments, demonstrate reading capabilities markedly inferior to those expected for their age and intellectual capacity, even when provided with sufficient educational resources (Lyon et al., 2003). Cross-linguistic studies reveal a consistent incidence rate of approximately 7.1% across both alphabetic and logographic languages (Yang et al., 2022). Contemporary research on dyslexia’s origins converges on two predominant theories: linguistic and non-linguistic specificity. Proponents of the linguistic specificity perspective argue that dyslexia’s core challenges originate from intrinsic deficits in processing and expressing linguistic information (Goswami et al., 2016), with notable impairments in phonological processing (Layes et al., 2015) and orthographic skills (Moura et al., 2020). Conversely, the non-linguistic specificity framework attributes dyslexia to underlying cognitive deficits, encompassing areas such as audiovisual perception (Ortiz et al., 2014), memory (Smith-Spark et al., 2016), and attentional control (Bosse et al., 2007), proposing a broader cognitive foundation for the disorder. Importantly, there is also a close connection between linguistic and non-linguistic processing, both of which can jointly influence reading (Cirino et al., 2022). In addition to the aforementioned theories, researchers have also discovered that both genetic and environmental factors interact dynamically in influencing the development of dyslexia, with genetics predisposing individuals to reading difficulties and environmental conditions, such as education and home literacy environment, either exacerbating or alleviating these challenges (Lyytinen et al., 2006).

In the context of DD, research on children with DD has consistently shown deficits in visual search (VS) performance (Guilbert and Rochette, 2023). Individuals with dyslexia often exhibit inaccuracies, such as repetitions and omissions, while reading. For example, Sireteanu et al. (2008) found that German children with DD (aged 8–19) had slower response times and lower accuracy in VS tasks compared to their chronological age-matched (CA) peers. Liu et al. (2019) corroborated the prevalence of VS deficits across different writing systems, indicating that these processing deficits are consistent among children with DD, irrespective of the writing system used. However, most studies focus on single age groups, neglecting variations in VS performance across different developmental stages. According to Wolf’s (2007) “Reading Brain” theory, the brain’s development plays a crucial role in forming reading abilities at various ages, and changes in brain structure and function during the early to late elementary school years directly impact reading fluency (RF) and comprehension. Therefore, it is essential to compare VS performance among children with DD across different age stages.

The reading process begins with the processing of visual graphic information, which is pivotal for literacy development. A growing body of research has explored the relationship between VS and reading abilities. For instance, Guilbert and Guiraud-Vinatea (2022) demonstrated a strong link between reading proficiency and VS accuracy. Plaza and Cohen (2006) found that 6-year-old French children who excelled in identifying target items among distractors showed better subsequent word reading and spelling abilities. A longitudinal study by Franceschini et al. (2012) identified VS and spatial cueing tasks as effective predictors of letter naming, RF, and reading accuracy among preschoolers during their first two grades, even after controlling for variables like age, intelligence quotient (IQ), and phonological awareness. Furthermore, VS has been recognized as a predictor of reading comprehension; for example, Solan et al. (2007) demonstrated that VS tasks could accurately identify a significant percentage of seventh-grade children with poor reading comprehension. Intervention studies, such as those by Solan et al. (2003), revealed that a 12-week VS training significantly improved reading comprehension in sixth-grade children with DD.

Research involving Chinese-speaking children is particularly important, as Chinese characters are ideographic and largely consist of phonetic and semantic radicals. Approximately 80% of Chinese characters incorporate semantic radicals that correlate with their meanings (Shu et al., 2006). This close form-meaning connection may render VS skills especially critical for Chinese reading comprehension. Liu et al. (2015) found that VS capacity uniquely predicts reading comprehension in typically developing children. Significant correlations have also been established between visual spatial attention and both vocabulary and text-level reading (Ding et al., 2016).

Numerous studies have confirmed the significant effect of VS on reading comprehension, including cross-sectional, longitudinal, and intervention studies. Increasingly, researchers have begun to investigate the mechanisms by which VS affects reading comprehension. Liu introduced the concept of “word detection skill” (WD), which assesses children’s ability to identify vocabulary boundaries within texts, positing that WD might mediate the relationship between VS and reading comprehension. While previous research has supported this hypothesis among typically developing children, it remains untested in children with dyslexia. Moreover, VS capabilities are closely related to RF in the reading process (Zhao et al., 2019). Proficient VS skills enable readers to quickly and accurately locate the next unit of reading, thereby enhancing automated decoding and comprehension. The efficiency of extracting textual information during reading directly affects both RF and comprehension levels. Research indicates that students with strong WD skills perform better in paragraph RF (Liu et al., 2022), and RF has been shown to mediate the relationship between VS and reading comprehension (Liu et al., 2024). Tong et al. (2023) highlighted that children’s lexical knowledge predicts sentence RF, ultimately affecting reading comprehension. However, while existing studies have examined the individual mediating roles of WD and RF, the chained mediating effect between these variables has not been thoroughly explored, particularly in children with dyslexia.

Given the increasing interest in understanding how VS deficits impact reading abilities, this study aims to address several gaps in the literature. We will investigate: (1) the differences in VS, WD skill, RF, and reading comprehension abilities among Chinese-speaking children with DD across second, fourth, and sixth grades; and (2) the chained mediating roles of WD skill and RF in the relationship between VS and reading comprehension.

## 2 Materials and methods

### 2.1 Participants

The study participants consisted of 191 Chinese-speaking children from second, fourth, and sixth grades. Among these, 92 children were diagnosed with dyslexia (*M* = 9.60 years, SD = 1.69 years; 45 females), including 29 s graders (*M* = 7.51 years, SD = 0.30 years; 18 females), 33 fourth graders (*M* = 9.60 years, SD = 0.26 years; 17 females), 30 sixth graders (*M* = 11.64 years, SD = 0.30 years; 10 females); and 99 children were CA typically developing children (*M* = 9.52 years, SD = 1.69 years; 42 females), including 33 s graders (*M* = 7.45 years, SD = 0.25 years; 12 females), 35 fourth graders (*M* = 9.62 years, SD = 0.29 years; 18 females), 31 sixth graders (*M* = 11.59 years, SD = 0.28 years; 12 females). The dyslexic children were identified through referrals from resource classrooms in their primary schools located in Hebei province. These individuals exhibited reading scores beneath the 25th percentile for their grade level, as determined by school-based assessments in the Chinese language. Furthermore, their performance on the Chinese Character Recognition Test (Li et al., 2012), a mandatory standardized test for the identification of learning disabilities within the cities from which participants were recruited, fell more than 1.5 SD below the mean.

The inclusion criteria for the study stipulated that children must demonstrate normal intelligence, measured by an IQ score above 85 on the Raven’s Progressive Matrices, and have no history of auditory impairments or psychiatric disorders, as assessed through teacher-completed questionnaires regarding each child’s health background. Dyslexic and control participants were closely matched in terms of age and gender. All children possessed either normal vision or vision that was corrected to normal. Reports from teachers and parents confirmed the absence of developmental disorders, including attention deficit hyperactivity disorder and dyspraxia, among the study participants. The Ethics Committee of Hebei Normal University approved this study, which complied with the Declaration of Helsinki. Informed consents were obtained from all participants’ parents and teachers.

### 2.2 Measures

#### 2.2.1 Raven’s standard progressive matrices

The Raven’s Standard Progressive Matrices were employed to evaluate children’s non-verbal intelligence. Children were required to select the most appropriate option from 6 to 8 choices to complete the target picture (Zhang and Wang, 1989). A total of 60 items were organized into 5 groups in ascending order of difficulty. Each correct answer was scored one point. The internal consistency coefficient for this task was 0.86.

#### 2.2.2 Visual search

**Figure 1:**
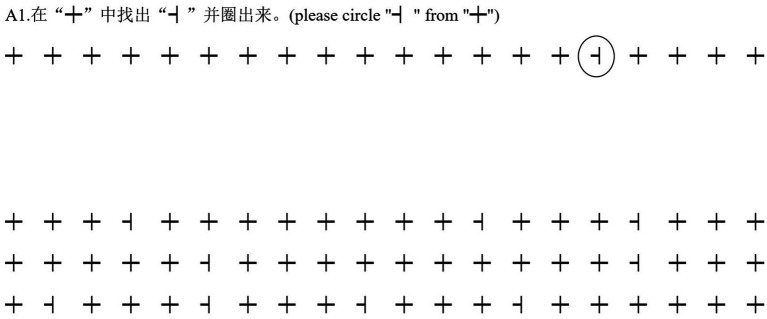
A sample trial in the visual search task.

The current study’s VS task was modified from the methodology of Liu et al. (2015, 2019). Participants were asked to circle target items among a number of distractors as accurately and as quickly as possible. The presentation of target items and distractors was randomized. Targets were categorized into three groups: symbols, alphabetic letters and characters, across a total of 24 trials. Each trial displayed 60 items in 3 rows, with each row containing 20 items. The count of target items fluctuated between 11 and 13 in different trials to prevent predictability of target quantity. Prior to the main circling activity in each trial, participants underwent a practice session with two lines of stimuli, including both targets and distractors, for acclimatization. The stimuli were displayed using Microsoft Jheng Hei Font at size 11. The participant’s completion time was recorded using a stopwatch in each trial. The number of errors were calculated by adding the number of target items that were missed and the number of distractors that were incorrectly circled. The following formula was used for indicating the performance in this visual search task: (number of accurately circled targets−number of errors)/completion time. The reliability of this measure was confirmed with a Cronbach’s alpha of 0.95. The specific target and distractor items utilized in this task are detailed in Table 1, with an example trial illustrated in Figure 1.

**Table 1:** All items used in the visual search task.

Item type	Item similarity							
	High-similarity pairs				Low-similarity pairs			
Symbol	┿:┥	↗:↙	♪:♩	∝:∽	△:□	⊕:□	≠:×	∑:×
Letter	O:Q	E:F	P:R	M:N	D:E	C:L	P:A	G:V
Character	牛:午	百:白	向:同	太:大	水:工	小:多	万:入	米:只

The right item in each pair is the target, while the left is the distractor.

#### 2.2.3 Word detection task

A WD task was developed to assess children’s performance in identifying two-character words in strings of characters (Liu et al., 2019). This task emphasizes the identification of multiple two-character words within a string of characters, rather than searching for a single target stimulus, as is typically the case in visual search task. There were 16 trials in total, with two rows in each trial. Participants were tasked with encircling bisyllabic words swiftly and precisely, moving from left to right and then top to bottom. Each line contained two words amid 16 non-target characters at random locations, ensuring the final character of the first line and the initial character of the second line did not constitute a word. Characters used were sourced from first-grade Chinese textbooks and presented in Microsoft Jheng Hei Font at size 16.

**Figure 2:**
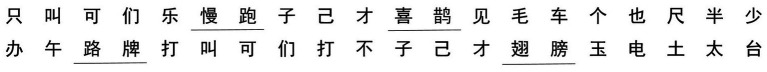
A sample trial in the word detection task. The target words are underlined.

The design of the WD task was a within-subjects factorial arrangement of 2 (visual complexity level: high versus low) by 2 (character categorization: target versus non-target), creating four distinct experimental conditions distributed across the trials, with each condition featuring four trials. Visual complexity, determined by stroke count, varied such that characters within the background and target words exhibited either high or low complexity levels. Characters of high complexity (Mean = 12.00, Standard Deviation = 0.88) contained a greater number of strokes compared to those of low complexity (Mean = 5.00, Standard Deviation = 0.70; *t* = 67.33, *p* < 0.001). For each experimental condition, the time taken to complete the task was logged, and performance inaccuracies were quantified by summing undetected target words and erroneously encircled non-words. An efficiency index for WD skill was computed to balance speed against accuracy, using the formula: efficiency = (number of correctly crossed out words−number of errors)/time. The reliability of this measure was confirmed with a Cronbach’s alpha of 0.87. Figure 2 depicts an example trial from this task.

#### 2.2.4 Chinese character reading

A Chinese character recognition task was deployed to measure the accuracy of word recognition. Children were presented with a sequence of 150 Chinese characters, arranged from the simplest to the most complex, and were asked to name them in order as accurately as possible without a specific emphasis on the speed of naming (Li et al., 2012). The test was terminated if a child made 15 consecutive naming errors or failed to respond. Each correctly named character was awarded one point, with the total number of correctly named characters constituting the final score for this task.

#### 2.2.5 Reading fluency

The RF test employed a three-minute silent reading task of sentences (Li et al., 2012), administered individually. The reading material consisted of 100 simple sentences, organized in ascending order of word count. The task required children to quickly read the sentences silently within 3 min and then then assess whether the content of the sentences was accurate. If a participant’s judgment was accurate, the score awarded was the number of words in that sentence; if incorrect, the score was 0. The total test score was the sum of all the words in the sentences judged correctly by the students.

#### 2.2.6 Reading comprehension

Reading comprehension tests designed for assessing the reading comprehension skills of children in Grades 2, 4, and 6, as noted by Wen (2005). This methodology is widely recognized and extensively used in research, as evidenced by studies conducted by Geng (2014) and Cheng et al. (2018). In the current study, children were presented with a written narrative and were required to answer both objective, multiple-choice questions and a subjective, simple question based on the text’s content (Geng, 2014). The first reading material, titled “Beautiful Little Flower Bed,” comprised a total of 277 Chinese characters, followed by 10 questions, including 9 single-choice and 1 open-ended question. The second material, “Beach Combing,” contained 382 Chinese characters, followed by 10 questions, mirroring the format of the first with 9 single-choice and 1 open-ended question. The third material, “In the Vegetable Garden,” consisted of 579 Chinese characters, followed by 10 questions, which included 8 multiple-choice and 2 open-ended questions. Children were expected to respond to the single-choice questions based on the content of the text, primarily evaluating their ability to obtain and understand the information presented. Each correctly answered multiple-choice question was awarded one point. For open-ended questions, evaluators assigned scores ranging from 1 to 3, based on the coherence with the text, the originality of the child’s interpretation, and the demonstration of divergent thinking in their responses. The cumulative total score for each participant was computed by aggregating the scores obtained from both categories of questions.

### 2.3 Procedure

All the tasks were administered individually by trained testers in quiet classrooms in one primary school during school time. The total testing time was about 1 h for each child. After the completion of all measures, each child was given a small gift for their participation.

### 2.4 Data analysis

Data were analyzed using SPSS version 27.0 to conduct variance analysis and correlation analysis. Initially, a series of ANOVAs were conducted with group (DD and CA) and grade level (2nd, 4th, and 6th) as between-subjects factors to analyze the dependent variables: VS, WD, RF, and reading comprehension with assumptions of normality and homogeneity of variances satisfied. Post-hoc comparisons were performed using Bonferroni adjustments to identify specific group differences when significant main effects or interactions were found. Furthermore, correlation analyses were performed to explore the relationships between VS, WD, RF, and reading comprehension. Pearson correlation coefficients were calculated to determine the strength and direction of these relationships. The significance level for all statistical tests was set at *p* < 0.05.

Finally, Model testing was also conducted using Amos version 24. Structural Equation Modeling (SEM) was employed to assess the relationships between VS, WD skill, RF, and Chinese reading comprehension, allowing for an exploration of direct and mediated effects. Model fit was evaluated with Chi-square, comparative fit index (CFI), Tucker-Lewis coefficient (TLI), and root-mean-square error of approximation (RMSEA), and standardized root mean square residual (SRMR). The model fit was considered reasonable when CFI and TLI were larger than 0.90 (Hu and Bentler, 1999), and RMSEA was less than 0.1 (Browne and Cudeck, 1993). Confidence intervals (CIs) (95% bias-corrected) were generated for the direct and indirect effects. If the CI of an effect did not include zero, we concluded that the indirect effect was different from zero, and thus was significant.

## 3 Results

### 3.1 Comparison of differences in various variables between CA and DD children

The analysis of VS showed a significant main effect of group (*F*(1, 185) = 6.82, *p* = 0.01, η^2^ = 0.04), and grade (*F*(2, 185) = 96.55, *p* < 0.001, η^2^ = 0.51). There was a significant interaction between group and grade (*F*(2, 185) = 5.22, *p* = 0.006, η^2^ = 0.05). Post-hoc comparisons with Bonferroni adjustment revealed that among 2nd graders, no significant disparities were evident in VS proficiency (*F*(1, 185) = 1.25, *p* = 0.27). Conversely, for 4th graders (*F*(1, 185) = 9.61, *p* = 0.002, η^2^ = 0.05) and 6th graders (*F*(1, 185) = 6.81, *p* = 0.01, η^2^ = 0.04), DD demonstrated significantly inferior VS outcomes compared to *CA*.

The ANOVA on scores of WD showed a significant main effect of group (*F*(1, 185) = 38.50, *p* < 0.001, η^2^ = 0.17), and grade (*F*(2, 185) = 62.53, *p* < 0.001, η^2^ = 0.40). The interaction between group and grade was not significant (*F*(2, 185) = 0.85, *p* = 0.43). These results suggested that DD had lower WD scores than CA in the 2nd, 4th, and 6th grades.

Regarding RF, substantial main effects were discerned for both group (*F*(1, 185) = 50.37, *p* < 0.001, η^2^ = 0.21) and grade (*F*(2, 185) = 110.72, *p* < 0.001, η^2^ = 0.55), with children facing dyslexia exhibiting significantly reduced fluency compared to peers and fluency scores escalating with grade progression. There was no significant interaction between group and grade (*F*(2, 185) = 2.84, *p* = 0.06). This indicated that dyslexic children were generally less accurate than typical children on RF.

**Figure 3:**
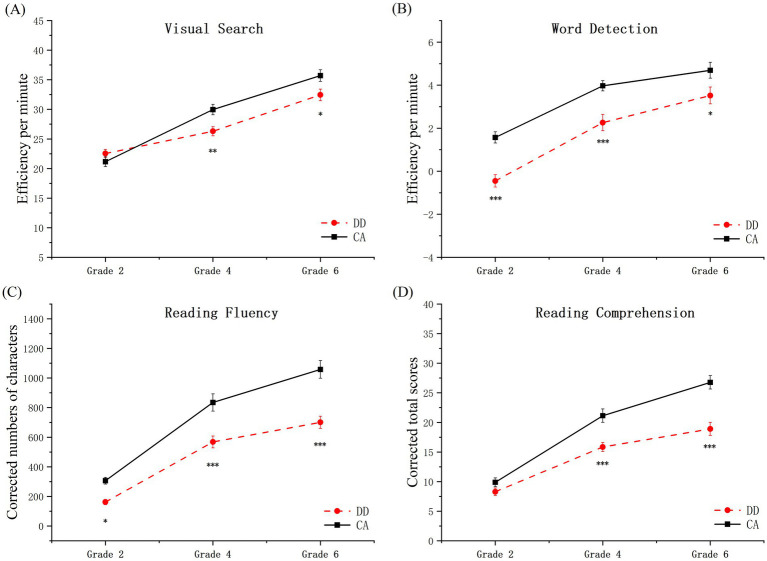
Scores of DD and CA groups of children on four tasks. (A) Visual search; (B) word detection; (C) reading fluency; (D) reading comprehension. Efficiency of visual search = (number of accurately circled targets−number of errors)/completion time. Efficiency of word detection = (total words identified - errors)/completion time. The score for reading fluency is the number of Chinese characters in the correctly judged sentences. The reading comprehension score reflects the points earned for correctly answering the questions. *p < 0.05, **p < 0.01, ***p < 0.001. The error bars represent the standard error.

The analysis of reading comprehension showed a significant main effect of group (*F*(1, 185) = 40.06, *p <* 0.001, η^2^ = 0.18), and group (*F*(2, 185) = 105.94, *p* < 0.001, η^2^ = 0.53). A notable interaction between group and grade was detected (*F*(2, 185) = 5.30, *p* = 0.006, η^2^ = 0.05). *Post-hoc* comparisons with Bonferroni adjustment revealed that no significant variance in reading comprehension among 2nd graders (*F*(1, 185) = 1.38, *p* = 0.24); however, considerable differences emerged in 4th grade (*F*(1, 185) = 16.54, *p* < 0.001, η^2^ = 0.08) and 6th grade (*F*(1, 185) = 32.85, *p* < 0.001, η^2^ = 0.15), with reading comprehension scores for DD being significantly below *CA.* Detailed findings are delineated in Table 2 and Figure 3.

**Table 2:** Descriptive statistics of all tasks.

Grade	Group	Visual search (M ± SD)	Word detection skill (M ± SD)	Reading fluency (M ± SD)	Reading comprehension (M ± SD)
2	*DD*	22.54 ± 3.78	−0.45 ± 1.54	161.72 ± 96.25	8.28 ± 3.45
	*CA*	21.15 ± 4.77	1.57 ± 1.47	306.09 ± 131.42	9.88 ± 4.28
4	*DD*	26.30 ± 4.45	2.26 ± 2.15	568.03 ± 231.82	15.85 ± 4.36
	*CA*	29.96 ± 5.19	3.97 ± 1.44	834.63 ± 346.88	21.14 ± 6.71
6	*DD*	32.44 ± 5.30	3.52 ± 2.12	700.50 ± 227.38	18.90 ± 6.09
	*CA*	35.70 ± 5.48	4.69 ± 2.05	1058.06 ± 333.69	26.77 ± 6.29

DD, developmental dyslexia, CA, chronological age-matched typically children. The score for visual search is calculated as (number of accurately circled targets − number of errors)/completion time. The score for word detection is calculated as (total words identified – errors)/completion time. The score for reading fluency is the number of Chinese characters contained in the correctly judged sentences. The reading comprehension score represents the points earned for correctly answering the questions.

### 3.2 Correlation analysis

The means and standard deviations for age and the measures, as well as the correlations between them, are summarized in Table 3. An analysis of skewness and kurtosis indicated that the data followed a normal distribution. Pearson’s correlation analysis revealed significant positive relationships among VS, WD skill, RF, and reading comprehension.

**Table 3:** Correlation among all variables of total children.

	Mean	SD	Skew	Kurt	1	2	3	4	5	6
1. Age (years)	9.56	1.69	−0.03	−1.38	-					
2. Raven	36.34	7.84	−0.26	−0.22	0.43**	-				
3. Visual search	28.00	7.06	0.36	0.09	0.70**	0.46**	-			
4. Word detection skill	2.63	2.46	−0.20	−0.04	0.56**	0.42**	0.52**	-		
5. Reading fluency	610.28	388.19	0.83	0.64	0.66**	0.41**	0.60**	0.63**	-	
6. Reading comprehension	16.89	8.24	0.39	−0.70	0.67**	0.55**	0.68**	0.64**	0.68**	-

**p < 0.01. Raven’s scores are used to assess non-verbal intelligence.

### 3.3 Effects of visual search on reading comprehension

We utilized AMOS 24.0 to conduct Structural Equation Modeling (SEM) to examine the relationships between VS, WD skill, RF, and Chinese reading comprehension. The primary aim of our model was to investigate how VS affects reading comprehension, both directly and indirectly, through the mediating roles of WD skill and RF. In our model, VS was hypothesized to directly influence reading comprehension, while WD skill and RF were modeled as mediators in this relationship. Specifically, we proposed that VS would enhance WD skill, which in turn would facilitate RF, ultimately leading to improved reading comprehension.

The measurement model confirmed that each observed variable significantly loaded onto its respective latent factor, indicating strong construct validity. Model fit was assessed using several indices: Chi-square, Comparative Fit Index (CFI), Tucker-Lewis Index (TLI), Root Mean Square Error of Approximation (RMSEA), and Standardized Root Mean Square Residual (SRMR). The goodness of fit of the model was indicated when CFI and TLI values exceed 0.95 and RMSEA was less than 0.06. The model fit was considered reasonable when CFI and TLI were larger than 0.90, and RMSEA was less than 0.08 (Hu and Bentler, 1999).

In our analysis, WD skill and RF were modeled as mediating factors through which VS exerts its effect on reading comprehension. Specifically, we posited that VS enhances WD skill, which subsequently influences RF, ultimately contributing to improved reading comprehension outcomes. We controlled for grade and Raven’s scores in the model. The model fit was reasonable, $x^{2}/df=2.358$ , SRMR = 0.063, TLI = 0.946, CFI = 0.961, RMSEA = 0.085. Although the value of RMSEA is greater than 0.08, according to the criteria set by Browne and Cudeck (1993), a model with an RMSEA less than 0.1 is still considered acceptable.

**Figure 4:**
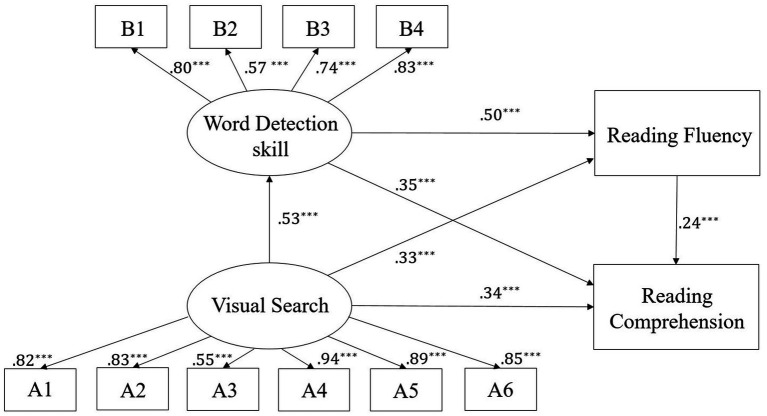
The relationship between visual search and reading comprehension. A1, High-similarity pairs of symbols; A2, High-similarity pairs of letters; A3, High-similarity pairs of characters; A4, Low-similarity pairs of symbols; A5, Low-similarity pairs of letters; A6, Low-similarity pairs of characters; B1, Low-complexity background with high-complexity targets; B2, High-complexity background with low-complexity targets; B3, Low-complexity background with low-complexity targets; B4, High-complexity background with high-complexity targets. ***p < 0.001.

The results demonstrated that VS had a significant direct effect on reading comprehension (95% CI [0.21, 0.44], *p* < 0.001). Additionally, we observed a significant indirect effect of VS on reading comprehension through WD skill (95% CI [0.10, 0.30], *p <* 0.001) and through RF (95% CI [0.03, 0.15], *p <* 0.001). Furthermore, the combined mediation effect of WD skill and RF between VS and reading comprehension was significant (95% CI [0.03, 0.12], *p <* 0.001). Overall, the model accounted for 66.5% of the variance in reading comprehension, highlighting the critical roles of both WD skill and RF in mediating the relationship between VS and reading comprehension. Details of the model are presented in Table 4 and illustrated in Figure 4.

**Table 4:** Direct and indirect effects and confidence intervals of models.

Rode	Effect size	SE	95% CI		
			Lower	Upper	*p*
VS → RC	0.34	0.06	0.21	0.44	<0.001
VS → WD → RC	0.19	0.05	0.10	0.30	<0.001
VS → RF → RC	0.08	0.03	0.03	0.15	<0.001
VS → WD → RF → RC	0.06	0.02	0.03	0.12	<0.001

VS, visual search; WD, word detection; RC, reading comprehension; RF, reading fluency.

## 4 Discussion

This study aims to compare the performance differences in VS, WD skill, RF, and reading comprehension tasks among DD and CA across different educational stages. The results indicate that in second graders, DD did not show any difference in VS tasks compared to their typically developing peers, contradicting earlier findings by Sireteanu et al. (2008). A possible explanation is that compared to alphabetic writing systems, Chinese reading begins later, and second graders, who are still at the initial stages of developing VS capabilities, did not exhibit differences from DD. However, by fourth grade, DD significantly underperformed in VS tasks compared to their typically developing peers, consistent with the findings of Liu et al. (2019). This suggests that as the learning difficulty and cognitive demands increase, the deficiencies in VS capabilities of DD begin to manifest, persisting into the sixth grade. These findings align with the multi-factorial view of dyslexia proposed by Bosse et al. (2007), indicating potential impairments in visual attention span and difficulties in attention maintenance and shifting in DD (Lallier et al., 2010). The developmental trajectory of VS capabilities with grade progression showed that both DD and CA improved over time, but between second and fourth grade, typically developing children demonstrated significantly faster progress, while DD improved more slowly, marking a critical turning point for the VS deficits in DD. This is closely related to the challenges in visual information processing speed and cognitive developmental stages, especially during the critical period from ages 3 to 8, which involves the transition from concrete to formal operational stages and the learning of symbolic systems such as text, shapes, and spatial relationships (Piaget, 2003). DD may have an inherent visual information processing speed impairment, limiting their ability to improve in VS tasks, especially in environments with increasingly demanding reading requirements (Regtvoort et al., 2006).

The relationship between visual search and reading is an area of growing interest, particularly regarding how visual processing skills contribute to reading development. Research, including studies by Franceschini et al. (2012), indicates that effective visual search capabilities may predict reading proficiency. In our study, we found that while no significant differences in visual search performance were observed between the groups in the 2nd grade, notable differences emerged in the 4th and 6th grades. This pattern suggests that as children gain reading experience, their visual search skills become more refined, enhancing their ability to quickly and efficiently locate relevant information within text. The development of reading skills may thus rely on the interplay between visual search and comprehension processes, where improved visual search capabilities facilitate quicker recognition of words and phrases, ultimately supporting reading fluency. Understanding this relationship is crucial, as it highlights the potential for targeted interventions aimed at improving visual search skills as a means to bolster reading outcomes in children.

In the aspect of WD skill, the study found that DD in second, fourth, and sixth grades performed significantly lower on WD tasks compared to *CA.* Although the WD skill of DD enhanced as they progressed through grades, their performance consistently lagged behind that of typically developing peers. Liu suggests that WD skill primarily reflects the ability to process visual information effectively during word recognition (Liu et al., 2019). Moreover, the study also indicates that DD perform worse in visual discrimination tasks within deep orthographic systems (such as Chinese) compared to shallow orthographic systems (like alphabetic writing), as observed by Serena et al. (2022). Regarding RF, the findings of this research are consistent with previous studies, showing that DD from second to sixth grade consistently scored lower on RF tests than their *CA.* This suggests a persistent cross-grade deficiency in RF among DD, a lasting developmental impairment that requires rich and targeted teaching strategies and support to improve their reading skills (Wolf and Bowers, 1999). In terms of reading comprehension, the study revealed a different trend. There is no significant difference between second-grade DD and their typically developing peers, but by fourth and sixth grades, their scores were significantly lower. According to the six-stage theory of reading development, the second to fourth grades are within the word decoding and RF stages, far from the development of reading at the discourse level, with typically developing children’s reading comprehension abilities also in a developmental stage (Chall, 1983). Hence, the significant differences between the two groups in WD and RF did not extend to reading comprehension. However, by fourth and sixth grades, as children’s reading development enters the multiple construct period, reading comprehension begins to rely on the deepening of integrated cognitive and reading skills, coupled with the increased complexity of reading materials and cognitive demands, leading to a significant deficiency in reading comprehension abilities among DD compared to CA (Chall, 1987).

VS capabilities are closely linked to reading comprehension abilities. VS not only directly impacts the level of reading comprehension but also exerts an influence on reading comprehension through intermediate links such as WD skill and RF (Franceschini et al., 2012; Liu et al., 2024). Both VS and WD tasks require individuals to acquire and parse information through the visual channel. The former focuses primarily on the process of locating and identifying target objects or features in complex scenes, involving attention allocation and pattern recognition, while the latter, based on the former, requires individuals to engage in cognitive activities related to reading comprehension and language processing. It is this underlying cognitive process, namely visual spatial attention that accounts for the close relationship between these two measures. Spatial attention is critical for early word recognition, involving cognitive processes such as focused attention, character scanning, and inhibition of irrelevant information, which are particularly crucial for reading Chinese characters (Besner et al., 2005). Good VS capabilities help children perform well in WD tasks, providing direct and indirect support for reading comprehension. Moreover, WD skill is closely related to RF and comprehension abilities. With the progression of grades and the accumulation of vocabulary knowledge, especially the increase in vocabulary depth, the impact on RF becomes more significant, thereby promoting the overall improvement of reading abilities (Liu et al., 2022; Verhoeven et al., 2011). This result can explain the phenomenon in this study where reading fluency differences between groups increase with higher grades. RF, as a factor independent of word decoding and comprehension, can directly affect the quality of reading comprehension (Hsu et al., 2023). Among higher-grade students, RF, especially sentence RF in silence, becomes a key indicator affecting reading comprehension, relying on the optimization of complex language information processing and attention allocation strategies (Samuels and LaBerge, 1974).

VS, WD, and RF collectively form a chain of mediating effects that influence reading comprehension. VS not only has a direct impact on reading comprehension but also enhances it indirectly by improving WD and RF. It plays a vital role in developing reading comprehension by enabling individuals to quickly locate and accurately capture textual information, thereby fostering WD skills and RF. Consequently, in teaching practices for DD, educators should prioritize the development of these three essential abilities: VS, WD, and RF. Implementing personalized teaching strategies is crucial, including specialized training to enhance VS, boost lexical recognition efficiency, expand vocabulary, and deepen understanding of words, ultimately providing a strong foundation for reading comprehension. While VS, WD, and RF are interconnected, their influences on reading comprehension must be considered within the specific context of the Chinese language. We acknowledge that these processes are measured indirectly and can be affected by multiple factors, which may influence the interpretation of our results. This clarification aims to provide a more nuanced and balanced perspective on the relationships among these variables, ensuring that our conclusions reflect the complexities inherent in reading comprehension processes, particularly in the context of Chinese literacy development. However, it is essential to acknowledge that different writing systems and cultural environments may significantly influence these developmental processes. Therefore, future research should focus on cross-cultural comparisons to further elucidate the relationship between visual search and reading comprehension.

In our study, we selected specific tasks and measures to assess visual search and reading comprehension based on their relevance to existing literature. The visual search task was consistent with those used in the research by Liu et al. (2015) and Liu and Chen (2019), which is widely recognized for assessing children’s visual search capabilities. However, a limitation of this task is its reliance on a paper-and-pencil format, which may confound task performance due to motor response demands, as participants were required to circle the targets. Additionally, the timed nature of the visual search may be influenced by general processing speed, potentially affecting results. Future research could benefit from implementing tasks that minimize motor responses and specifically examine the search performance of dyslexic children with and without processing speed impairments. Despite these concerns, we believe these confounding factors do not significantly threaten the validity of our findings, as they likely impact both efficient and inefficient searches similarly, with impairments mainly observed in inefficient searches. Furthermore, this study did not include a control group matched to the reading level of dyslexic group, which could help isolate dyslexia-specific effects. Future research could further clarify the causal role of VS by incorporating a control group matched on reading level. Finally, the reading comprehension task utilized in this study follows the methodology established by Wen (2005), which has been validated in studies by Geng (2014) and Cheng et al. (2018). Our findings indicate that this task poses considerable challenges for second-grade children, which may lead to floor effects. Therefore, future research should not only utilize generally applicable reading comprehension materials but also incorporate grade-appropriate texts to align better with the developmental levels of the participants.

In summary, this study underscores the significance of visual search capabilities in reading comprehension, not only for DD but also for typically developing readers. The findings suggest that difficulties in efficiently identifying key information can hinder comprehension across diverse populations, including those with varying levels of decoding proficiency. Given the complexity of reading comprehension, it is crucial to consider the impact of environmental factors and instructional quality on the development of these skills. As noted, different orthographic systems present unique challenges that may influence how comprehension is learned and assessed. Therefore, future research should further investigate how visual search interacts with comprehension processes across various cultural contexts and reading environments. By expanding our understanding of these relationships, we can develop targeted interventions and instructional strategies that support all learners, ultimately fostering a greater appreciation for reading and enhancing literacy outcomes across diverse educational settings.

## Data Availability

The raw data supporting the conclusions of this article will be made available by the authors, without undue reservation.

## References

[ref1] Besner D. Risko E. F. Sklair N. (2005). Spatial attention as a necessary preliminary to early processes in Reading. Can. J. Exp. Psychol. 59, 99–108. doi: 10.1037/h0087465 16035344

[ref2] Bosse M.-L. Tainturier M. J. Valdois S. (2007). Developmental dyslexia: the visual attention span deficit hypothesis. Cognition 104, 198–230. doi: 10.1016/j.cognition.2006.05.009, PMID: 16859667

[ref3] Browne M. W. Cudeck R. (1993). “Alternative ways of assessing model fit” in Testing structural equation models. eds. Bollen K. A. Long J. S. (New York: Sage Publications), 136–162.

[ref4] Chall J. S. (1983). Stages of reading development. New York: McGraw-Hill, 10–24.

[ref5] Chall J. S. (1987). Reading and early childhood education: the critical issues. Special report: early childhood education. Principal 66, 6–9.

[ref6] Cheng Y. Wang J. Wu X. (2018). The role of morphological awareness in Chinese children’s reading comprehension: the mediating effect of word reading fluency (in Chinese). Acta Psychol. Sin. 50:413. doi: 10.3724/SP.J.1041.2018.00413

[ref7] Cirino P. T. Barnes M. A. Roberts G. Miciak J. Gioia A. (2022). Visual attention and reading: a test of their relation across paradigms. J. Exp. Child Psychol. 214:105289. doi: 10.1016/j.jecp.2021.105289, PMID: 34653633 PMC8608740

[ref8] Ding Y. Zhao J. He T. Tan Y. Zheng L. Wang Z. (2016). Selective impairments in covert shifts of attention in Chinese dyslexic children. Dyslexia 22, 362–378. doi: 10.1002/dys.1541, PMID: 27805322

[ref9] Franceschini S. Gori S. Ruffino M. Pedrolli K. Facoetti A. (2012). A causal link between visual spatial attention and Reading acquisition. Curr. Biol. 22, 814–819. doi: 10.1016/j.cub.2012.03.013, PMID: 22483940

[ref10] Geng Y. (2014). Measurement and intervention research on morphological awareness in children with developmental Reading difficulties in Chinese. Xiamen, Fujian: Xiamen University.

[ref11] Goswami U. Cumming R. Chait M. Huss M. Mead N. Wilson A. M.. (2016). Perception of filtered speech by children with developmental dyslexia and children with specific language impairments. Front. Psychol. 7:791. doi: 10.3389/fpsyg.2016.00791, PMID: 27303348 PMC4885376

[ref12] Guilbert A. Guiraud-Vinatea H. (2022). Links between organized visual search and reading ability in French primary school children. Dyslexia 28, 97–109. doi: 10.1002/dys.1703, PMID: 34820936

[ref13] Guilbert A. Rochette F. (2023). Visual search organization in a cancellation task in developmental dyslexia. Cogn. Neuropsychol. 40, 148–157. doi: 10.1080/02643294.2023.2286026, PMID: 38105578

[ref14] Hsu L. S.-J. Chan K. Ho C. S.-H. (2023). Reading fluency as the bridge between decoding and reading comprehension in Chinese children. Front. Psychol. 14:1369. doi: 10.3389/fpsyg.2023.1221396 PMC10497962 37711329

[ref15] Hu L. Bentler P. M. (1999). Cutoff criteria for fit indexes in covariance structure analysis: conventional criteria versus new alternatives. Struct. Equ. Model. 6, 1–55. doi: 10.1080/10705519909540118

[ref16] Lallier M. Tainturier M. J. Dering B. Donnadieu S. Valdois S. Thierry G. (2010). Behavioral and ERP evidence for amodal sluggish attentional shifting in developmental dyslexia. Neuropsychologia 48, 4125–4135. doi: 10.1016/j.neuropsychologia.2010.09.027 20933526

[ref17] Layes S. Lalonde R. Mecheri S. Rebaï M. (2015). Phonological and cognitive Reading related skills as predictors of word Reading and Reading comprehension among Arabic dyslexic children. Psychology 6, 20–38. doi: 10.4236/psych.2015.61003

[ref18] Li H. Shu H. McBride-Chang C. Liu H. Peng H. (2012). Chinese children’s character recognition: visual-orthographic, phonological processing and morphological skills. J. Res. Read. 35, 287–307. doi: 10.1111/j.1467-9817.2010.01460.x

[ref19] Liu D. Chen X. (2019). Visual search and reading comprehension in Chinese children: the mediation of word detection skill. Read. Writ. 33, 1163–1182. doi: 10.1007/s11145-019-09996-x

[ref20] Liu D. Chen X. Chung K. K. H. (2015). Performance in a visual search task uniquely predicts Reading abilities in third-grade Hong Kong Chinese children. Sci. Stud. Read. 19, 307–324. doi: 10.1080/10888438.2015.1030749

[ref21] Liu S. Li N. Zhang X. Wang L.-C. A. Liu D. (2024). Effects of working memory and visual search skill on Chinese reading comprehension: examining the simple view of reading. Read. Writ. 4:10515. doi: 10.1007/s11145-024-10515-w

[ref22] Liu S. Wang L. C. Liu D. (2019). Deficits of visual search in Chinese children with dyslexia. J. Res. Read. 42, 454–468. doi: 10.1111/1467-9817.12277

[ref23] Liu D. Xu Z. Wang L.-C. (2022). The interaction between morphological awareness and word detection skills in predicting speeded passage Reading in primary and secondary school Chinese readers. Front. Psychol. 13:802005. doi: 10.3389/fpsyg.2022.802005, PMID: 35310202 PMC8927659

[ref24] Lyon G. R. Shaywitz S. E. Shaywitz B. A. (2003). A definition of dyslexia. Ann. Dyslexia 53, 1–14. doi: 10.1007/s11881-003-0001-9

[ref25] Lyytinen H. Erskine J. Tolvanen A. Torppa M. Poikkeus A.-M. Lyytinen P. (2006). Trajectories of reading development: a follow-up from birth to school age of children with and without risk for dyslexia. Merrill-Palmer Q. 52, 514–546. doi: 10.1353/mpq.2006.0031

[ref26] Moura O. Pereira M. Moreno J. Simões M. R. (2020). Investigating the double-deficit hypothesis of developmental dyslexia in an orthography of intermediate depth. Ann. Dyslexia 70, 43–61. doi: 10.1007/s11881-020-00190-1 32096102

[ref27] Ortiz R. Estévez A. Muñetón M. Domínguez C. (2014). Visual and auditory perception in preschool children at risk for dyslexia. Res. Dev. Disabil. 35, 2673–2680. doi: 10.1016/j.ridd.2014.07.007, PMID: 25063906

[ref28] Piaget J. (2003). Cognitive development in children: Piaget. J. Res. Sci. Teach. 40, S8–S18. doi: 10.1002/tea.3660020306

[ref29] Plaza M. Cohen H. (2006). The contribution of phonological awareness and visual attention in early reading and spelling. Dyslexia 13, 67–76. doi: 10.1002/dys.330, PMID: 17330736

[ref30] Regtvoort A. G. F. M. Leeuwen T. H. V. Stoel R. D. Leij A. V. D. (2006). Efficiency of visual information processing in children at-risk for dyslexia: habituation of single-trial ERPs. Brain Lang. 98, 319–331. doi: 10.1016/j.bandl.2006.06.006, PMID: 16870246

[ref31] Samuels S. LaBerge D. (1974). Toward a theory of automatic information processing in Reading. Cogn. Psychol. 6, 293–323. doi: 10.1016/0010-0285(74)90015-2

[ref32] Serena P. Barbara C. David G. AnneMarie A. Lorena M. Daniel R. (2022). Shallow or deep? The impact of orthographic depth on visual processing impairments in developmental dyslexia. Ann. Dyslexia 72, 171–196. doi: 10.1007/S11881-021-00249-7, PMID: 35286579 PMC8942915

[ref33] Shu H. McBride-Chang C. Wu S. Liu H. (2006). Understanding Chinese developmental dyslexia: morphological awareness as a core cognitive construct. J. Educ. Psychol. 98, 122–133. doi: 10.1037/0022-0663.98.1.122

[ref34] Sireteanu R. Goebel C. Goertz R. Werner I. Nalewajko M. Thiel A. (2008). Impaired serial visual search in children with developmental dyslexia. Ann. N. Y. Acad. Sci. 1145, 199–211. doi: 10.1196/annals.1416.021, PMID: 19076398

[ref35] Smith-Spark J. H. Zięcik A. P. Sterling C. (2016). Self-reports of increased prospective and retrospective memory problems in adults with developmental dyslexia. Dyslexia 22, 245–262. doi: 10.1002/dys.1528 27121331

[ref36] Solan H. A. Shelley-Tremblay J. Ficarra A. Silverman M. Larson S. (2003). Effect of attention therapy on Reading comprehension. J. Learn. Disabil. 36, 556–563. doi: 10.1177/00222194030360060601 15493437

[ref37] Solan H. A. Shelley-Tremblay J. F. Hansen P. C. Larson S. (2007). Is there a common linkage among reading comprehension, visual attention, and magnocellular processing? J. Learn. Disabil. 40, 270–278. doi: 10.1177/00222194070400030701, PMID: 17518218

[ref7001] Tong X. Chiu M. M. Tong S. X. (2023). Synergetic effects of phonological awareness, vocabulary, and word reading on bilingual children’s reading comprehension: A three-year study. Contemp. Educ. Psychol. 73 102153–25. doi: 10.1016/j.cedpsych.2023.102153

[ref38] Verhoeven L. van Leeuwe J. Vermeer A. (2011). Vocabulary growth and Reading development across the elementary school years. Sci. Stud. Read. 15, 8–25. doi: 10.1080/10888438.2011.536125

[ref39] Wen H. (2005). Primary scholar’ Chinese reading ability scales development, validity, and reliability. Guangzhou: South China Normal University.

[ref40] Wolf M. (2007). Proust and the squid: the story and science of the reading brain. New York, NY: Harper.

[ref41] Wolf M. Bowers P. G. (1999). The double-deficit hypothesis for the developmental dyslexia. J. Educ. Psychol. 91, 415–438. doi: 10.1037/0022-0663.91.3.415

[ref42] Yang L. Li C. Li X. Zhai M. An Q. Zhang Y.. (2022). Prevalence of developmental dyslexia in primary school children: a systematic1 review and meta-analysis. Brain Sci. 12:240. doi: 10.3390/brainsci12020240, PMID: 35204003 PMC8870220

[ref43] Zhang H. C. Wang X. P. (1989). Standardization research on Raven’s standard progressive matrices in China. Acta Psychol. Sin. 21, 113–121.

[ref44] Zhao J. Liu H. Li J. Sun H. Liu Z. Gao J.. (2019). Improving sentence reading performance in Chinese children with developmental dyslexia by training based on visual attention span. Sci. Rep. 9:18964. doi: 10.1038/s41598-019-55624-7, PMID: 31831849 PMC6908582

